# Supplementary material for: Adeno-Associated Virus (AAV)-Delivered Exosomal TAT and BiTE Molecule CD4-αCD3 Facilitate the Elimination of CD4 T Cells Harboring Latent HIV-1
Source: Microorganisms. 2024 Aug 18;12(8):1707. doi: 10.3390/microorganisms12081707 (PMC11357122; doi:10.3390/microorganisms12081707)
Supplement: Supplementary file 1 [file microorganisms-12-01707-s001.zip › microorganisms-3161364-supplementary.pdf]

Supplementary Figure S1

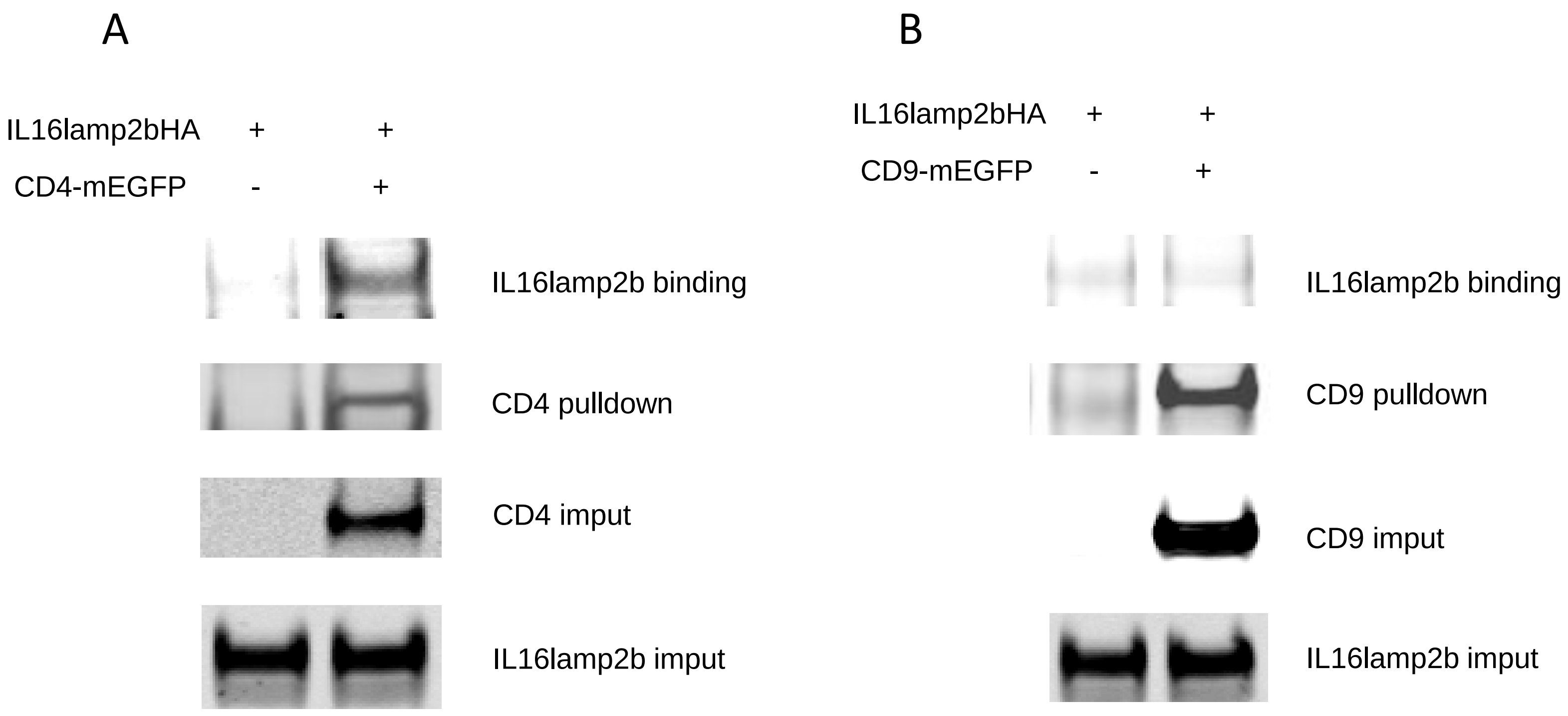

Figure S1. IL16lamp2b specifically binds to CD4 but not CD9. Expression plasmids of IL16lamp2b, CD4-mEGFP or CD9-mEGFP was transfected into HEK293T cells respectively using Lipofectamine 2000. Forty-eight hours post-transfection, cell lysates of transfected cells were prepared using Pierce IP/Lysis Buffer and used for immunoprecipitation/western blot assays as indicated in the figure. A: IL16lamp2b specifically binds to CD4. IL16lamp2b lysate was mixed with HEK293T control lysate or CD4-mEGFP lysate. Anti-GFP magnetic beads were used to pull down CD4. Anti-HA mAb was used to detect IL16lamp2bHA. B: IL16lamp2b doesn't bind to CD9. IL16lamp2b lysate was mixed with HEK293T control lysate or CD9-mEGFP lysate. Anti-GFP magnetic beads were used to pull down CD9. Anti-HA mAb was used to detect IL16lamp2bHA.

Supplementary Table S1

List of primers used for molecular cloning

Primer name	Sequence
ClaI-MES-f	AACATCGATATGGTGTGCTTCCGCCTCTTCCCGGTTCCG
ClaI-CD4-f	AACATCGATATGAACCGGGGAGTCCCTTTTAG
antiCD3-BglII-r	CGTAGATCTTTACTTGATTTCCACTTTGGTGCCTC
SalI-Exo-Tat-s	CTAGAGTCGACATGGGCTGCATTAACAGC
HA-BglII-r	CGTAGATCTTCATGCATAGTCCGGGACGTCATAG
HA-Furin-F2A-up-as	CAGCTTGAGAAGATCGAAGTTGAGAGTCTGCTTGACAGG AGCACGCTTAGCACGGTCCAACTTGAC
F2A-down-s	TCGATCTTCTCAAGCTGGCTGGTGATGTCGAGTCTAATC CTGGACCTATGGTGTGCTTCCGCCTC
Lamp2b-BglII-r	CGTAGATCTTTAAGCGTAATCTGGCACATCG
HA-Furin-F2A-up2-as	ATTTTAACAAGTCAAAATTTAACGTTTGTTTAACCGGTGCC CTTTTGGCCCTAGCGTAATCTGGCACATC
Middle-s	GCAGCATCTACTTATTCAATTGACAGCGTCTCATTTTC
Middle-as	GAAAATGAGACGCTGTCAATTGAATAAGTAGATGCTGC
